# Genome Sequences for Three Strains of Kocuria rosea, Including the Type Strain

**DOI:** 10.1128/genomeA.00594-18

**Published:** 2018-06-21

**Authors:** Ariel M. Trachtenberg, Abigail E. Goen, Kyle S. MacLea

**Affiliations:** a Biology Program, University of New Hampshire, Manchester, New Hampshire, USA; b Biotechnology Program, University of New Hampshire, Manchester, New Hampshire, USA; c Department of Life Sciences, University of New Hampshire, Manchester, New Hampshire, USA

## Abstract

Genomes from three strains of Kocuria rosea were sequenced. K. rosea ATCC 186, the type strain, was 3,958,612 bp in length with a total G+C content of 72.70%. When assembled, K. rosea ATCC 516 was 3,862,128 bp with a 72.82% G+C content. K. rosea ATCC 49321 was 4,018,783 bp in size with a 72.49% G+C content.

## GENOME ANNOUNCEMENT

The *Actinobacteria* include a large number of terrestrial and aquatic bacteria, including important soil microbes and pathogens. Kocuria rosea is a Gram-positive actinobacterium originally appreciated as a pigmented environmental isolate (1, 2) and later noted as an underappreciated human pathogen (3–10), potential bioremediator in the elimination of azo dyes and other chemicals (11), and producer of important compounds (12). Several strains of K. rosea were originally known under other basonyms (1, 13, 14) but are now grouped together in the same genus and species. To verify these taxonomic assignments to the K. rosea species and to provide a platform for comparison among the environmental, commensal, and pathogenic strains of the species, a sequencing project was undertaken to genomically evaluate three distinct strains of K. rosea.

K. rosea ATCC 186^T^, ATCC 516, and ATCC 49321 were obtained from the ATCC (Manassas, VA, USA) in lyophilized form and rehydrated. Cultures were grown at 26°C on nutrient or brain heart infusion medium from isolated colonies of each strain, with careful attention on keeping each strain pure and separate. The QIAamp DNA minikit (Qiagen, Valencia, CA, USA) was used to isolate genomic DNA (gDNA) from single colonies grown in broth culture according to the manufacturer’s instructions. Purified gDNA was fragmented, adapter tagged using the Nextera DNA library prep kit (Illumina, San Diego, CA, USA), and sequenced with an Illumina HiSeq 2500 instrument at the Hubbard Center for Genome Studies (Durham, NH, USA). The resulting 250-bp paired-end read sequences were bioinformatically trimmed using Trimmomatic (15) prior to assembly and gene analysis.

Assembly and annotation of each genome were undertaken using SPAdes v. 3.11.1 and the NCBI Prokaryotic Genome Annotation Pipeline (PGAP) process (16). Type strain ATCC 186 had a genome size of 3,958,612 bp with a G+C content of 72.70%. The other strains had similar sizes and G+C contents; K. rosea ATCC 516 was 3,862,128 bp with a G+C content of 72.82%, and strain ATCC 49321 was 4,018,783 bp in size with a G+C content of 72.49%. A total of 3,564 to 3,718 genes were identified in each of the three strains by the PGAP. RNA genes, including rRNA, tRNAs, noncoding (ncRNAs), and 84 to 100 pseudogenes were found in each strain. No clustered regularly interspaced short palindromic repeat (CRISPR) arrays were found. A total of 162 nonidentical reductases were found in the three strains, consistent with the noted observation of high K. rosea reductase activity in decolorization and detoxification of azo dyes, which is of potential use in cleanup and bioremediation (11). Comparisons among the strain sequences using average nucleotide identity (ANI) on the EzBioCloud portal (17) support their assignment to the same species, as each strain had greater than 98% ANI compared with the other two strains in any dyad combination. The genome sequences generated in this study should prove useful in the comparison of these strains with other K. rosea isolates with environmental, commensal, and pathogenic origins.

### Accession number(s).

The Kocuria rosea whole-genome shotgun (WGS) projects were deposited under the GenBank accession numbers QFBJ00000000 (ATCC 186^T^), QFBK00000000 (ATCC 516), and QFBL00000000 (ATCC 49321). The versions described in this paper are the first versions, QFBJ01000000, QFBK01000000, and QFBL01000000, respectively.
